# Refining humane endpoint detection by time-series forecasting and threshold definition using a multivariate severity score

**DOI:** 10.3389/fphys.2026.1869563

**Published:** 2026-07-08

**Authors:** Sara Lutscher, Lisa Goral, Christine Häger, Anna Munk, Miriam Heider, Nora Weegh, Dietmar Zechner, Heidrun Potschka, Andreas von Knethen, André Bleich, Steven R. Talbot

**Affiliations:** 1 Institute for Laboratory Animal Science, Hannover Medical School, Hannover, Germany; 2 Institute of Pharmacology, Toxicology, and Pharmacy, Ludwig-Maximilians-Universität München, Munich, Germany; 3 Rudolf-Zenker-Institute for Experimental Surgery, Rostock University Medical Center, Rostock, Germany; 4 Department of Anesthesiology, Intensive Care Medicine and Pain and Therapy, University Hospital Frankfurt, Frankfurt, Germany; 5 Institute of Medical Statistics, RWTH Aachen University Hospital, Aachen, Germany

**Keywords:** 3Rs, forecasting, humane endpoint prediction, preclinical research, refinement, severity assessment

## Abstract

Severity assessment in animal-based research is not only mandatory but also requires the identification of robust, objective, and model-specific parameters. The RELative Severity Assessment (RELSA) procedure enabled multivariate severity assessment, allowing interpretation of outcomes in the context of a predefined severity grading. This study expands the application of the RELSA score by combining it with an Autoregressive Integrated Moving Average (ARIMA)-based prediction model, which we call foRcast. To facilitate interpretation of severity, a kernel density estimate (KDE) was applied to define exploratory thresholds on the RELSA scale. Data of mice reaching humane endpoints across models representing a broad range of severity profiles were reanalyzed using transmitter-derived parameters (e.g., heart rate) and behavioral parameters (e.g., voluntary wheel running), depending on the model. The primary focus was predicting RELSA scores at the humane endpoint using foRcast. With an overall root mean square error of 0.069 and 96% of actual RELSA scores falling within the 95% prediction interval of the forecasts, this tool showed promising preliminary performance. KDE was performed on sepsis data, yielding candidate thresholds at RELSA = 0.337 and RELSA = 0.647, which could be used to define severity zones for classifying the animals’ burden, although these should not be confused with regulatory severity gradings. This proof-of-concept study showed the potential of the foRcast tool to accurately predict RELSA scores across various animal models and interventions, demonstrating the possibility to identify individual animals at risk of reaching the humane endpoint. The data also emphasize the intrinsic limitation of ARIMA models: drastic changes in the course cannot be reliably predicted. This underscores the need for higher-resolution measurements to detect early changes in animal well-being. Overall, the definition of candidate severity thresholds on the RELSA scale and the foRcast model represent valuable additions to the range of severity tools for refining severity assessment in animal-based research.

## Introduction

The assessment of the severity of procedures in animal-based research is mandatory under the EU directive 2010/63/EU (EU Commission, 2010) but still needs further development (De Vleeschauwer et al., 2023; Smith et al., 2018). Identifying robust, model- or intervention-specific parameters is crucial for reliably monitoring animal welfare and interpreting findings appropriately in the context of the animal model under consideration (Flecknell, 1994; Keubler et al., 2020; Pinkernell et al., 2016). From an ethical perspective, a precise severity assessment is required, among other things, to prevent prolonged suffering and to identify humane endpoints (Morton, 2023; Talbot et al., 2020). This need for proper welfare assessments is underscored by the proposed refinement of the 3R principle by Russell and Burch (Russell and Burch, 1959), which is well established in the scientific community and in European legislation (Bleich et al., 2023). Additionally, the reproducibility of procedures depends on accurate severity assessments (Bleich et al., 2020; Jirkof et al., 2020; Kahnau et al., 2020), as high-quality research relies on good animal welfare (Poole, 1997). This is particularly important considering the ongoing reproducibility crisis (Kilkenny et al., 2009). As a first approach to an objective, multivariate severity assessment, the RELative Severity Assessment (RELSA) score was introduced. Instead of evaluating each measure in insolation, this procedure combines multiple parameters into a single, interpretable value. RELSA does so, by capturing how far the animal’s condition deviates from its normal state and relates this deviation to reference data with a defined severity, enabling interpretation of the endured burden (Talbot et al., 2022). So far, RELSA has been used to compare burden within and between different animal models, yielding this procedure as a robust severity assessment tool (Schreiber et al., 2024; Talbot et al., 2023, 2026). In animal experiments, various outcome measures are evaluated, including body weight, clinical score, and behavioral parameters (Kumstel et al., 2020; Tang et al., 2020). However, suboptimal parameter selection can lead to vague, subjective, or potentially inaccurate welfare monitoring (Zieglowski et al., 2020).

Therefore, we are introducing the foRcast tool to predict the RELSA and other time-dependent parameters throughout experimental procedures. Forecasting is widely used across scientific fields (Zellner et al., 2021), but, to the best of our knowledge, remains largely unexplored in the context of severity assessment. So far, humane endpoints have been estimated solely from changes in body weight using Bayesian time-series forecasting (Blaß et al., 2025).

The Autoregressive Integrated Moving Average (ARIMA) model used in this work is one of the most established mathematical frameworks in time-series forecasting (Reddy et al., 2025). This algorithm combines an autoregressive (AR) model with a moving-average (MA) model. The first predicts future values based on past observations, while the latter accounts for errors in prior measurements when making its predictions. The integrative part of the ARIMA model is included to satisfy the assumption of stationarity by differentiating the underlying data (Box et al., 2016; Feigelson et al., 2018; Liu et al., 2016).

The foRcast tool is intended for an individual, continuous severity assessment, based on previous measurements and observations of each animal, to predict the animal’s further development. As distress and burden are individually experienced impairments (Dawkins, 1990; Wemelsfelder, 1997), a single-animal assessment is needed for a thorough evaluation and an individual reduction of suffering. A particular interest lies in detecting animals at humane endpoint thresholds or at risk of exceeding the approved severity classification, so they receive special attention from handling personnel. The focus of the foRcast tool is to identify animals in critical conditions with a predicted recovery to prevent too-early euthanasia, while not prolonging unnecessary suffering in case of non-recovery. In combination with the RELSA procedure and model-specific parameters, the potential benefit of this approach would be a differentiated, objective, and comprehensive welfare overview, with the opportunity to introduce new humane endpoint definitions.

Further, the RELSA score of an individual animal requires the severity context of a reference set with defined categorical severity to understand the endured burden. So, a definition of thresholds on the RELSA scale is needed to interpret a given score directly within ordinal severity classes. Therefore, we use kernel density estimation (KDE) to estimate the population probability density function from the kernel distributions of a sample (Węglarczyk, 2018; Zambom and Dias, 2013). Thresholds are then defined by identifying minima in the function as areas of low occurrence (Gilles and Heal, 2014). These data-driven candidate thresholds indicate severity classes on the RELSA scale, enabling straightforward and comparable interpretations of distress.

Hence, we hypothesize this work yields a proof-of-concept of our ARIMA-based forecasting tool to accurately and robustly predicts changes in the animals’ well-being across different animal models. Moreover, we hypothesize that using the comprehensive RELSA score in forecasting provides better insights into severity assessment than single parameters, thereby enabling researchers to identify animals at risk of deteriorating health. Lastly, we hypothesize that the KDE algorithm can define exploratory thresholds on the RELSA scale for practical interpretation and for evaluating distress associated with the score.

## Materials and methods

### Data

In this study, previously published data from seven distinct animal models and interventions were reanalyzed. These included a cecal ligation and puncture (CLP) surgery model to induce sepsis, a dextran sulfate sodium (DSS)-induced colitis model with either restraint stress or with repeated facial vein blood sampling, a model of pancreatic cancer by injecting ductal pancreatic adenocarcinoma cells, and a neurosurgical intervention. Ethical statements were included in the original manuscripts. All animals underwent thorough clinical scoring, and humane endpoint criteria were applied to humanely euthanize animals based on predetermined clinical conditions. Clinical scoring differed between laboratories and models, and is therefore not directly comparable.

In the sepsis model, the data of two animals that reached the humane endpoint were used in the foRcast evaluation. For KDE, all animals in this study were considered, including the two mice that reached the humane endpoint, two mice surviving the sepsis, and three sham-operated animals. Before the CLP surgery, transmitters were implanted in the lateral flank between the fore- and hindlimbs, and the animals recovered completely. We considered the transmitter-derived parameters heart rate (hr [bpm]), heart rate variability (hrv [ms]), body temperature (temp [°C]), and activity (act [counts]) in the RELSA score calculations. The first three measures were averaged hourly, while the activity was summed up hourly. Humane endpoints were reached when >25% temperature loss was recorded by personnel over more than two consecutive, predefined monitoring intervals. Transmitter-derived parameters were analyzed retrospectively only (Talbot et al., 2022).

The mice in the DSS-colitis and restraint stress models received intraperitoneal transmitter implants, and the experimental procedures began when the animals were fully recovered. Here, the mice were treated with 1.5% DSS in drinking water for 5 days and placed in restraint tubes for 1 hour per day for 10 consecutive days. We analyzed data from two euthanized mice that reached humane endpoint criteria, using the same telemetric parameters as in the sepsis model, with the addition of body weight change (bwc [%]) in the foRcast evaluation. Again, in the KDE procedure the data of all animals of this study were used, resulting in four additional surviving mice. The parameters were averaged (bwc, hr, hrv, temp) or summed up (act) daily. Humane endpoints were reached at 20% body weight loss (Talbot et al., 2022).

In the DSS-induced colitis model with repeated facial vein blood sampling, the animals received either 1% or 1.5% DSS via the drinking water. Facial vein phlebotomy was additionally performed on six of the seven animals considered in the foRcast evaluation on days 0, 5, and 14. Daily assessments of the bwc [%] and voluntary wheel running (vwr [%]) in seven euthanized mice that reached humane endpoint criteria were analyzed using the RELSA procedure. Humane endpoints were reached at either 20% body weight loss or a total clinical score of 5. Clinical score evaluation is described in the original publication. The surviving mice in this experiment were additionally considered in KDE calculations, including seven mice receiving 0% DSS, eight mice receiving 1% DSS without blood sampling, seven mice receiving 1.5% DSS without blood sampling, eight mice receiving 0% DSS with blood sampling, ten mice receiving 1% DSS with blood sampling, seven mice receiving 1.5% DSS with blood sampling, and lastly eight mice receiving 0% DSS with additional restraint stress (Häger et al., 2018). Additionally, in this model the data of voluntary wheel running was missing on the day of euthanasia. These observations were omitted to preserve the multivariate property of the RELSA procedure. Therefore, the last complete observation, i.e. the day before the humane endpoint criterion was met, was considered in the foRcast evaluation, henceforth this time point was referred to as pre-humane endpoint.

Pancreatic cancer was induced by injecting cells of the 6606PDA cell line into the pancreas. A single male mouse in the vehicle group that reached the humane endpoint was investigated in foRcast calculations using daily bwc [%] and vwr [%]. The humane endpoint was reached at 20% body weight loss (Weegh et al., 2021).

In the neurosurgical intervention study, mice underwent implantation of an intracranial electrode in the right amygdala under different analgesic treatment protocols. A single male mouse treated with carprofen monotherapy reached humane endpoint criteria, was euthanized, and was thus investigated for foRcast evaluation. The parameters bwc [%], nesting score (nesting), and Neuro Score (modified Irwin Score; neuro), which were measured daily, were used to calculate the RELSA scores. The humane endpoint was reached at a total clinical score of 7, which is further described in the original publication (Munk et al., 2024).

A comprehensive table of dataset information is in **Supplementary Material S1**. Further experimental details are found in the respective articles.

### Data analyses

All analyses were conducted with the R programming language in RStudio (R version 4.4.1 “Race for Your Life”) with the packages dplyr, tidyr, ggplot2, ggbeeswarm, RELSA, forecast, purrr, patchwork, effsize, readxl, stringr and lubridate.

### RELSA procedure

The RELSA score calculation comprises four steps. First, the outcome measures and their “directionality” must be chosen, which was done individually for each model. The default interpretation assumes that a decrease in a parameter indicates a worsening outcome. If a worsening is considered when a parameter increases, its directionality must be reversed (“turned”). In the DSS and restraint stress model, a worsening was expected with a rising heart rate and temperature, so these parameters were “turned” in the calculation. The same was done for the nesting behavior and Neuro Score for the neurosurgical intervention. All outcome measures considered for each animal model were already described. Secondly, the measurements need to be normalized, which was done in relation to individual baseline values in this study. The third step is defining a reference set. In this study, the animal in the treatment group suspected to experience the greatest burden under the respective models was used as the reference, e.g., the highest DSS dose with phlebotomy in the DSS and blood sampling dataset. Lastly, RELSA weights were calculated for each variable at each time point as the deviation of the outcome measure from the normalized baseline value, relative to the maximum deviation in the reference set. These weights were then combined using a root-mean-square operation to yield the final RELSA score. Further details are explained in the original publication (Talbot et al., 2022).

The outcome measures used in the RELSA procedure per model, the “turned” variables, baseline window, and reference group are provided in **Supplementary Table S2**.

### Kernel density estimation

Kernel density estimation reconstructs the probability density function underlying a sample. For each data point a kernel distribution is calculated from the observations with a defined bandwidth and where the kernel is, e.g., Gaussian (Ledl, 2016; Rosenblatt, 1956). The probability of a new data point is then calculated as the average of the probabilities from all the kernel distributions. Expanding this across the range of all values yields the overall probability density function, which allows inference to the population (Chen, 2017; Parzen, 1962). Identifying minima in the latter function enables the definition of low-occurrence areas, which can serve as thresholds (Korneev et al., 2022).

In this study, base R was used to perform KDE analysis using the density function. In the animal models considered, data from surviving animals and those that reached the humane endpoint criteria were included in this analysis.

### ARIMA-based foRcast model

The ARIMA model consists of the autoregressive and moving-average components. The first predicts future values based on previous time points and the number of lags, or the so-called order *p*, which defines the number of prior observations used for the predictions (Schaffer et al., 2021). In the MA model the predictions are based on the random errors of *q* previous time points, which are assumed to be white noise (Ivanovski et al., 2018; Schaffer et al., 2021). To conclude the ARIMA model, *d* differentiating steps are taken until the variance of the underlying data is removed, achieving stationarity and the absence of trends (Adhikari and Agrawal, 2013).

The R package forecast was used as the basis for our foRcast tool. The automatic ARIMA function systematically selects the preferred values for *p*, *d*, and *q* by computing four initial models and then minimizing the Akaike information criterion (AIC). The parameters *p* and/or *q* of the models with the lowest AIC are then varied in a specified range in search for a better AIC. This step is repeated until the lowest, and therefore best AIC is found (Hyndman and Khandakar, 2008). As experiments in animal-based research usually consist of one measurement per parameter per day over a limited experimental duration, a linear regression model interpolated time points at 0.1-day increments before ARIMA forecasting. This was done to provide foRcast with sufficient data, addressing the sparse data problem in “one measurement per day” experiments.

To evaluate the tool’s performance, we calculated three error metrics. The root mean square error (RMSE) calculated the square root of the mean squared error of the predictions and, therefore, quantified the deviation of the predicted values from the actual value (Mehdiyev et al., 2016), while the prediction interval coverage probability (PICP) depicted the percentage of actual values inside the prediction intervals of the forecasts (Pang et al., 2018). Contextualizing PICPs with the width of the prediction interval is necessary to avoid underestimated uncertainty, which was done by computing the mean prediction interval width (MPIW) (Khosravi et al., 2011). All measurements up to the time point immediately before the humane endpoint were used in the foRcast model to predict the RELSA score at the humane endpoint for each animal.

Since any relevant time-series (measurement variable) can be incorporated into the foRcast model, we examined the accuracy of predicting various outcome measures and RELSA scores. For that, two additional evaluation approaches were conducted in the sepsis model. This model was chosen because it represented the highest assumed severity and had the most data points and objective parameters. First, all outcome measures (i.e., heart rate, heart rate variability, temperature, activity, and RELSA scores) were forecast at each time point, and the relative deviations of predicted values from actual values were calculated. The second approach compared a direct RELSA score prediction to an indirect prediction. The former refers to forecasting RELSA scores as is, while the latter refers to predicting the outcome measures and then calculating the RELSA score from these predictions.

## Results

### The foRcast tool reveals accurate humane endpoint predictions

**Figure 1:**
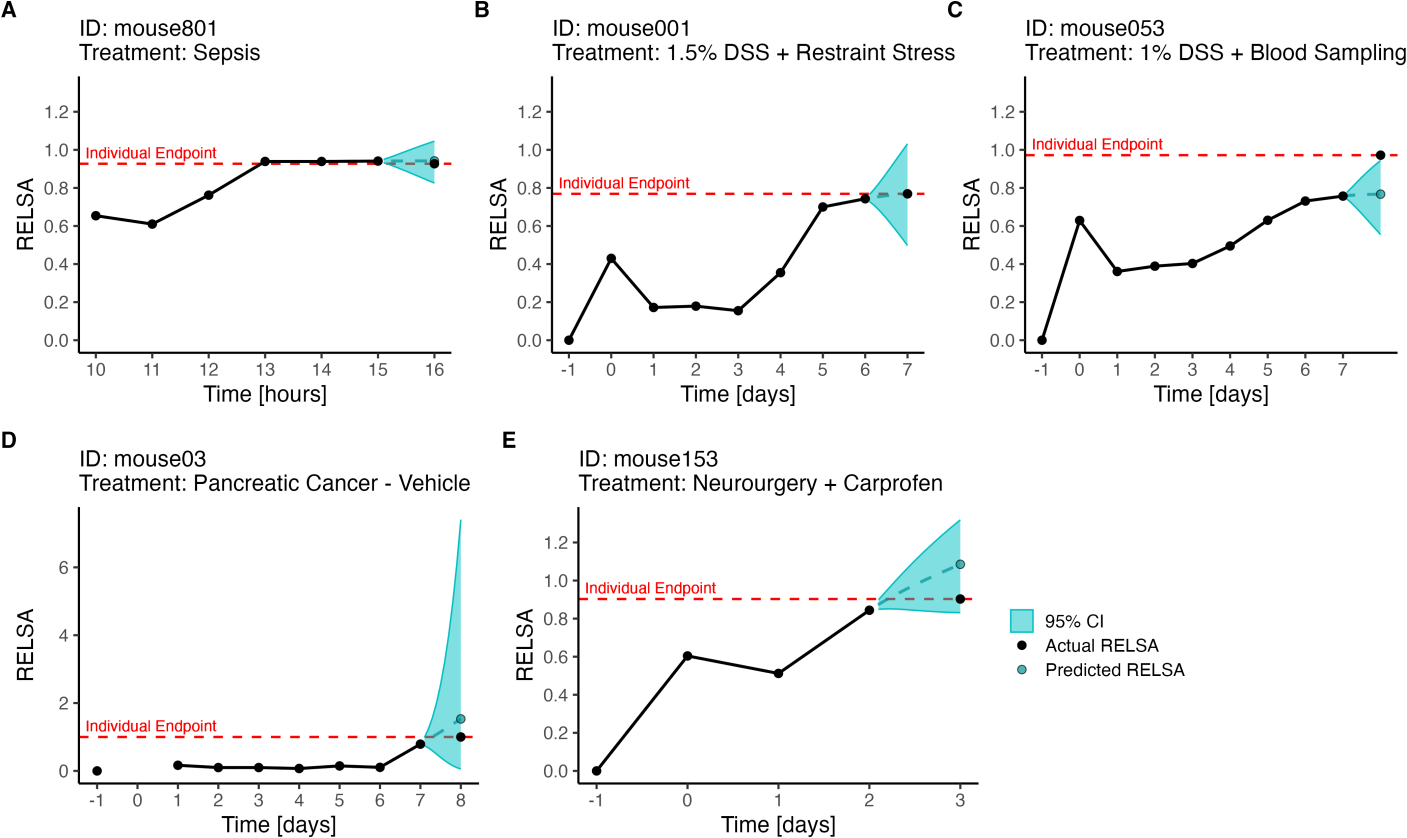
Individual RELSA score prediction of the (pre-)humane endpoint of exemplary mice in the different models and interventions. The red dashed lines depict the RELSA scores at the humane endpoint time point. The black data points show the actual RELSA scores, while the blue data points represent the humane endpoint prediction. The blue ribbons outline the 95% prediction intervals of the forecasts. **(A)** The foRcast prediction of the RELSA score at the humane endpoint for the mouse in the sepsis model. The previous time points of this animal (-1–9 hours) were omitted for illustrative purposes. **(B)** The foRcast prediction of the RELSA score at the humane endpoint for the mouse in the DSS model with restraint stress. **(C)** The foRcast prediction of the RELSA score at the pre-humane endpoint in the mouse DSS model with blood sampling. **(D)** The foRcast prediction of the humane endpoint for the mouse in the pancreatic cancer model. Mind the different y-axis scaling. **(E)** The foRcast prediction of the RELSA score at the humane endpoint for the mouse in the neurosurgical intervention.

RELSA scores were calculated using telemetry-derived parameters in the sepsis and DSS models with restraint stress, while behavioral parameters were considered in the remaining models. The predictions of humane endpoints in the sepsis model, the DSS model with restraint stress, and the neurosurgical intervention all showed high precision, with the actual RELSA scores of all animals in these models falling within the 95% prediction intervals of the forecasts (**Figures 1A, B, E**). Sepsis mouse 801 stabilized at the RELSA score of its humane endpoint starting 13 hours post-CLP surgery and underwent euthanasia 16 hours post-surgery (**Figure 1A**). The predefined manual measurement time points in this study were set at 8 and 16 hours post-surgery, indicating the humane endpoint at the latter time point. Only the retrospective analysis of hourly transmitter data showed that hypothermia was reached between the measurements. The prediction of the pre-humane endpoint of the mouse in the DSS model with blood sampling deviated more from the actual RELSA score, with the latter falling outside the 95% prediction bounds (**Figure 1C**). Although the actual RELSA score at the humane endpoint for the mouse in the pancreatic cancer model was well inside the 95% prediction interval of the forecast, the predicted score deviated more from the actual value than in the other models. Due to the width of the prediction interval, the y-axis had to be adjusted accordingly and differed from the others (**Figure 1D**).

The forecasting results of the other mice in the different models and interventions are illustrated in **Supplementary Figures S2**–**S5**. The trends of the individual outcome measures per mouse and study are illustrated in **Supplementary Figures S8**–**S12**.

Quantitative evaluation of the foRcast tool yielded an overall RMSE of 0.069, indicating a precise prediction (**Table 1**). The slightest deviation was seen in the DSS model with additional restraint stress (RMSE = 0.007), while the prediction for the single animal of the pancreatic cancer model depicted the greatest deviation (RMSE = 0.177). The PICP of 96% indicated precise forecasts, with perfect coverage across six of the seven models, although in three of these models, only one mouse was considered (**Table 1**). In the 1% DSS model with phlebotomy, only three of the four predicted pre-humane endpoints fell within their forecast prediction intervals, yielding the lowest PICP of 75%. Overall, MPIW reached 1.69, meaning that the prediction interval covered 169% of the RELSA range on average. The highest MPIW was observed in the pancreatic cancer model at 7.35, while the sepsis model showed the narrowest interval at 0.30 (**Table 1**).

**Table 1:** Performance evaluation of the foRcast function with root mean square error, prediction interval coverage probability, and mean prediction interval width per animal model and overall for mice that reached the (pre-)humane endpoint.

Animal model/intervention	No. of animals	RMSE	PICP [%]	MPIW
Sepsis	2	0.009	100	0.30
1.5% DSS + Restraint Stress	2	0.007	100	0.66
1% DSS + Blood Sampling	4	0.046	75	0.53
1.5% DSS + Blood Sampling	2	0.065	100	0.84
1.5% DSS	1	0.095	100	1.64
Pancreatic Cancer	1	0.177	100	7.35
Neurosurgery	1	0.082	100	0.54
Overall	13	0.069	96	1.69

### RELSA forecasting balances stability and variability across parameters

**Figure 2:**
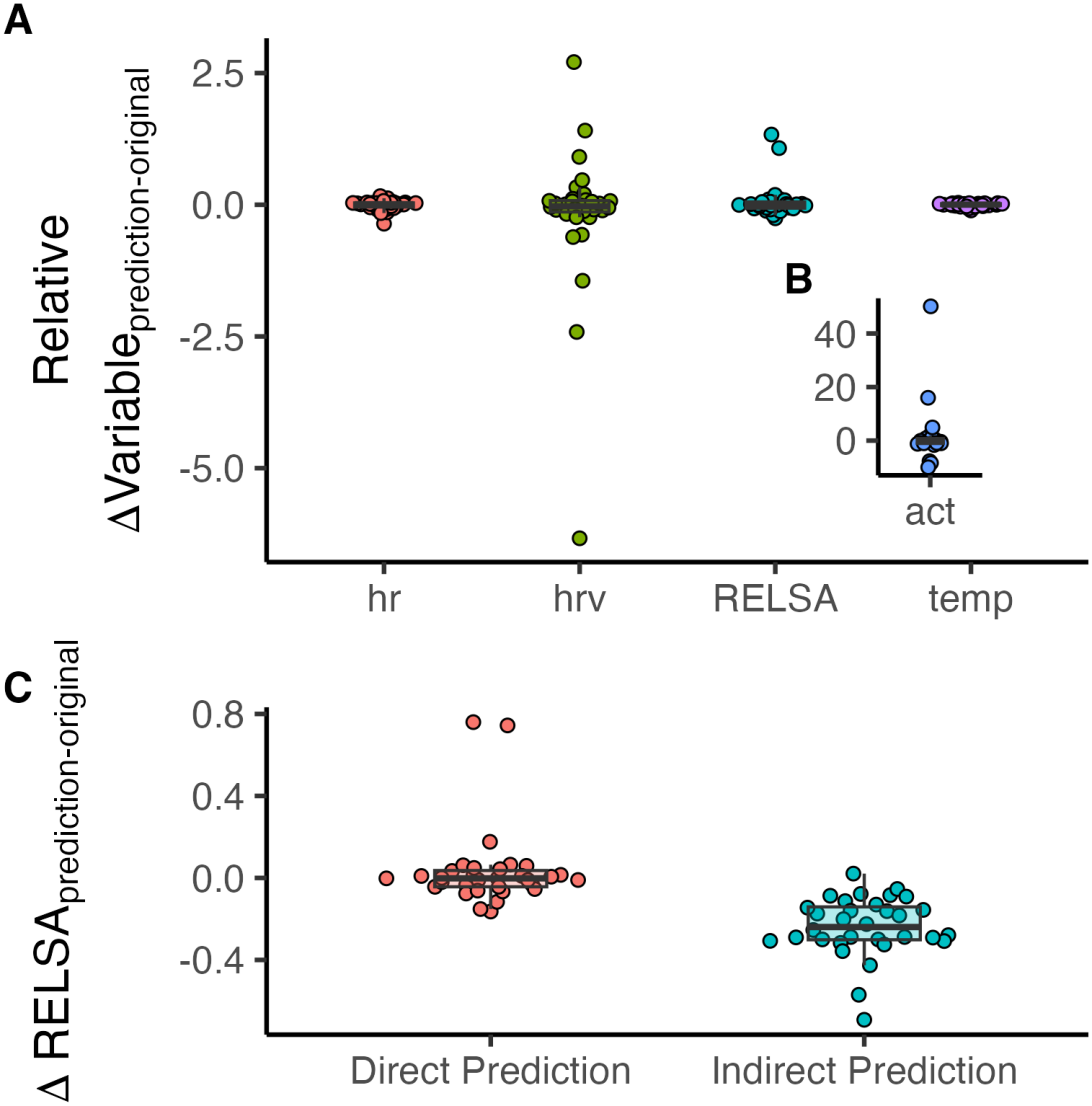
Comparison of the prediction performance for different outcome measures and direct and indirect RELSA score predictions in the sepsis model, coming from cecal ligation and puncture surgery. **(A, B)** All outcome measures and RELSA scores for mice that reached the humane endpoint were forecast at each time point, and the relative difference between the actual and predicted values was calculated. Each data point represents a single comparison. Boxes depict the interquartile range with the median as the line inside the box. Median deviations ranged between -0.065 and 0.022. The predictions for the heart rate and temperature showed the least differences. RELSA score predictions were also close to the actual values, although two predicted scores were higher than their counterparts. The predicted heart rate variability values deviated in both directions, whereas the forecast activity values showed the greatest variation. **(C)** RELSA scores were either forecast directly or indirectly via predicted outcome measures. Again, each data point represents the difference between actual and predicted values. Boxes depict the interquartile range with the median as the line inside the box. The directly predicted RELSA scores only differed slightly from the actual values, with a median deviation of -0.002. In contrast, the indirectly predicted RELSA scores reached a median deviation of -0.240. The difference between these groups revealed a large effect size [d = 1.42; 95% CI (1.03, 1.81)].

Predictions of all outcome measures and the RELSA score in the sepsis model were compared to further evaluate foRcast performance using two approaches. In the first approach, all parameters and RELSA scores of the animals reaching the humane endpoint were forecast at each time point, and the deviation between actual values and predictions was calculated. Here, the sepsis model parameters showed a median fluctuation of Δ = -0.065 to 0.022, with exceptionally stable predictions for heart rate and temperature. The forecasts for the RELSA score showed a similar pattern, with two predictions higher than the original values. The forecasts of heart rate variability showed greater variability in both directions, whereas the predictions of activity showed the greatest deviations (**Figures 2A, B**). In the second evaluation approach, RELSA score forecasting was compared with forecasting the outcome measures and calculating RELSA scores from these forecasts. The direct prediction of RELSA scores showed a median deviation of -0.002, with two higher predicted scores. The indirect predictions showed a median difference of -0.240, resulting in a large effect size of *d* = 1.42 (95% CI [1.03, 1.81]) between the two RELSA prediction methods (**Figure 2C**).

### Kernel density estimation allows the definition of severity zones on the RELSA scale

**Figure 3:**
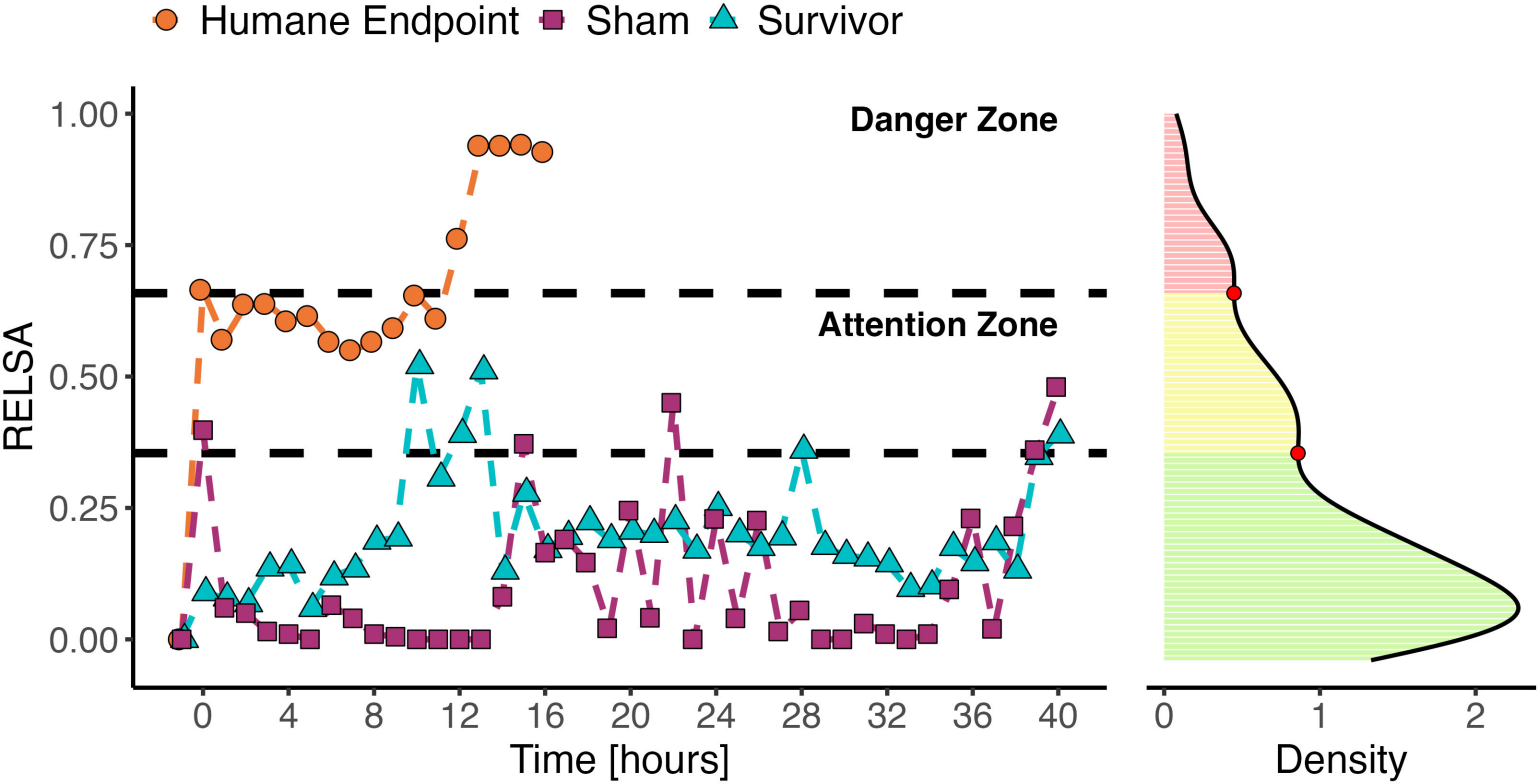
Definition of thresholds on the RELSA scale through kernel density estimation in the sepsis model. Each data point shows the calculated RELSA score for an individual, exemplary animal at each time point. The plot on the right shows the density curve with the two calculated minima at RELSA = 0.337 and RELSA = 0.643. These minima were used to divide the RELSA scale into sections, with an attention zone between the thresholds and a danger zone above the upper bound. While the surviving and sham-operated mice were mainly below the attention-zone threshold, the mouse that reached the humane endpoint quickly entered the upper end of the attention zone, with RELSA scores extending into the danger zone up to the humane endpoint. Data from all animals in the sepsis study were used in this analysis (n = 7 mice, 239 data points).

Kernel density estimation was performed to identify possible thresholds on the RELSA scale with data from all animals in the sepsis dataset. For illustrative purposes, the development of a single animal from each subgroup, namely sham-operated, surviving, and humane endpoint, was randomly selected. This analysis revealed candidate thresholds at RELSA = 0.337 and RELSA = 0.643, which we used to define model-specific, preliminary attention and danger zones. These do not represent the regulatory severity gradings set out in the EU Directive 2010/63/EU. As our prior investigations were conducted at the individual-animal level, the development of each animal was followed over time for each experimental condition. The sham-operated animal mostly stayed within the attention zone, although some RELSA scores slightly exceeded the threshold. The surviving mouse also had RELSA scores mostly below the attention zone, yet above those of the sham-operated animal. When the animal surpassed the threshold into the attention zone, its RELSA scores remained lower than those of the mouse which reached the humane endpoint. The latter quickly reached scores at the upper end of the attention zone, then entered the danger zone and subsequently the humane endpoint (**Figure 3**).

Because only a single mouse reached the humane endpoint in these studies, the pancreatic cancer model and neurosurgical intervention were excluded from this analysis. The KDE results for both DSS models under different stressors are shown in **Supplementary Figures S6** and **S7**.

## Discussion

Because animals are unable to express their distress directly (Kanzler et al., 2016), it is the ethical responsibility of researchers to develop reliable methods to assess animal suffering in research settings (Kiani et al., 2022). The RELSA procedure paved the way for a multivariate severity assessment with contextually interpretable results and was also successfully translated into a clinical application (Ohland et al., 2024). As the experienced burden is multifaceted (Helmig and Wenzel, 2024), we decided to use the RELSA tool as a multivariate approach to assess severity and predict humane endpoints. So, this study aimed to prove the conceptual validity of the foRcast approach and evaluate its performance, enabling its implementation as a prediction tool that expands RELSA’s applicability.

With an overall RMSE of 0.069, the RELSA scores at the humane endpoint were predicted reasonably accurately, as suggested by 96% of the actual scores falling within the 95% prediction intervals of the forecasts. The foRcast tool was shown to be applicable across various animal models, as indicated by precise predictions of performance metrics. In the single prediction for the pancreatic cancer model, a deviation of approximately 0.2 between the predicted and actual RELSA scores was observed. Usually, a rise of 0.2 in RELSA does not indicate a drastic change in the animal’s well-being, although a threshold could be surpassed if those were defined in the respective model. In the event of an overestimated RELSA score, this would simply encourage personnel to handle the animal with special care. However, an underestimated RELSA score could discourage personnel from giving the individual the necessary attention and increase the risk of delaying humane endpoint decisions. Therefore, it is essential to note that the RELSA procedure is intended as an aid to severity assessment rather than a decisive parameter, so animals with a lower RELSA score that exhibit other signs of distress should still be handled accordingly. To provide a more nuanced interpretation of the predicted RELSA score, the coverage of the prediction interval and its width should be considered. With a PICP of 96%, the actual values were reliably within the prediction bounds. Importantly, increased uncertainty leads to larger prediction intervals (Hazra, 2017), increasing the likelihood that the actual RELSA scores fall within the prediction bounds and consequently increasing the PICP. The MPIW was additionally considered to put the PICP into perspective with the width of the prediction intervals. While the forecast value in the pancreatic cancer model was well within the prediction interval, the latter spanned 735% of the RELSA range, undermining the reliability of the PICP. This higher uncertainty may be attributable to the relatively constant development of the RELSA score in this mouse, with a drastic increase at the time point just before the humane endpoint (**Figure 1D**). However, when combined, all performance metrics indicate that the foRcast tool provides valid, consistent predictions across different models. Nevertheless, the small sample size of 13 mice should also be considered. As this is a proof-of-concept study for the foRcast function, more data, preferably from different experimental and laboratory contexts, need to be incorporated for generalized validity and performance statements. Exploration of models in which the animals’ conditions worsen, with subsequent recovery, may also pose interesting foRcast domains.

The pre-humane endpoint for a mouse in the DSS model with blood sampling was not correctly predicted (**Figure 1C**). This deviation occurred because the RELSA score at the pre-humane endpoint increased markedly compared with prior observations for this animal, highlighting the ARIMA model’s intrinsic limitation in predicting abrupt changes. This structural constraint stems from the mathematical foundation of the ARIMA construct, which assumes stationarity and linearity, rendering it unable to predict changes when the variance is unequal (Petrică et al., 2016). Additionally, the automatic ARIMA function of the forecast package was used in the foRcast tool, which explores a defined range of values for *p*, *d*, and *q* to find the best ARIMA model (Hyndman and Athanasopoulos, 2021). The overall best model could potentially be outside of this predefined range of values; however, the general performance of the foRcast model provides adequate results.

Moreover, we assume a rapid deterioration in the animal’s well-being between the last two data points, which may be better predicted by a higher measurement frequency (Qin et al., 2019) or alternative forecasting models allowing modeling of sudden fluctuations, like Bayesian online changepoint detection or Markov switching models (Adams and Mackay, 2007; Hamilton, 2020). On top of that, the considered prediction time point was the day before the humane endpoint criterion was met, as the data of voluntary wheel running was missing on the day of euthanasia. This was done to preserve the multivariate characteristic of the RELSA score. A complete observation at the day of euthanasia could potentially have depicted a better forecasting result, as the drastic change that occurred at the pre-humane endpoint may have influenced the general trajectory of the animal’s development. Beyond improving predictive accuracy, high-resolution, continuous measurement may also advance animal welfare monitoring, as demonstrated by the retrospective transmitter data analysis in the sepsis study (**Figure 1A**).

While the minimum number of data points required to achieve reliable forecasting results was originally and universally set at 50 (Box et al., 2016), this number has recently been challenged (Hassouna and Al-Sahili, 2020). In this work, high-resolution measurements were unavailable, and to address the general sparse-data problem in animal experimentation, the foRcast function included an interpolation step between observed measurements. As this increases autocorrelation and partial autocorrelation, both of which are used by the automatic ARIMA function to identify model parameters, the performance of the function with and without these additional values was compared. This evaluation identified interpolation as a necessary alteration of the original ARIMA function to minimize errors while maximizing prediction interval coverage with narrower boundaries. Usually, animal experiments covering acute models with “one measurement per day” designs cannot provide sufficient data for time-series forecasting. But with the pending implementation of continuous monitoring data, the interpolation step will be redundant, allowing the use of native ARIMA forecasting in our foRcast function.

A comprehensive table including all performance metrics across both model adaptations.

When comparing the predictive ability of the foRcast tool across the different outcome measures in the sepsis mouse model, activity predictions performed the worst (**Figure 2B**). We concluded from this analysis that some parameters are more predictable than others, which may result from their inherent volatility, leading to greater noise (Chodakowska et al., 2021) in combination with low-frequency measurements. However, using multiple variables in the RELSA procedure can temper the impact of a single parameter’s characteristic, yielding better predictive performance without information loss. The direct prediction of the RELSA score outperformed the indirect prediction, as expected. In indirect prediction, the errors of single-parameter forecasts accumulate and propagate when calculating the RELSA score (Selim et al., 2020).

In contrast, the direct prediction carries only the error of the predicted RELSA score itself. Generally, the differences between the predicted and actual values are gathered around zero. Yet, because the foRcast tool is designed at the single-animal level, individual deviations must be considered. In the direct prediction of RELSA scores, two larger differences, Δ = 0.76 and Δ = 0.74, were calculated (**Figure 2C**). These are large differences on the RELSA scale and would change the distress estimation. Both predictions occurred at the earliest possible time point, so only two measurements were used in these forecasts. With this limited prior knowledge in the foRcast model, a reliable prediction is understandably difficult (Abeysinghe et al., 2003). As stated before, the tool incorporates linear regression to increase the number of data points used for predictions. This was done to ensure sufficient data were available for the model, but could be removed with higher-frequency measurements. Here, implementing automated home cages with high-resolution measurements of objective parameters, such as heart and respiratory rates, would greatly benefit the foRcast and RELSA procedures (Mösch et al., 2023).

With the implementation of the RELSA procedure, a tool for multivariate severity assessment was introduced. At the same time, there is a need for thresholds on the RELSA scale to interpret the values more subtly in terms of animal welfare. The EU directive mandates a prospective assessment across four categories: non-recovery, mild, moderate, and severe (EU Commission, 2010), and a generalized RELSA scale with severity classifications could be an appropriate instrument to help researchers determine the appropriate category. The kernel density estimation delivered thresholds at RELSA = 0.337 and RELSA = 0.643, which were used to define distinct zones of distress in the sepsis model (**Figure 3**). Nevertheless, these thresholds are neither generalizable nor directly translatable into severity categories under EU Directive 2010/63/EU. They are also limited to this particular model due to the RELSA procedure’s relativity and the lack of comparable animal models. We also investigated the two DSS-induced colitis models. In the DSS and restraint stress model, a threshold at RELSA = 0.250 was calculated, while one at RELSA = 0.649 was found for the DSS model with blood sampling (**Supplementary Figures S6**, **S7**). Although these are fairly close to both thresholds in the KDE analysis of the sepsis model, again, these thresholds are not comparable to each other and are not generalizable. Nevertheless, they do show a general area on the RELSA scale where common thresholds may lie. As the minima in the KDE probability density function are needed to define thresholds, we assume that the number or distribution of data points is responsible for the differing results in the considered models (Chen et al., 2018). Again, both the pancreatic cancer model and neurosurgical intervention were excluded from this analysis due to sparse data in the higher-value ranges. Larger datasets, through the use of automated home cages or higher-frequency measurements, could also help refine this analysis.

This study presented a proof-of-concept for the foRcast tool as a promising predictor of the humane endpoint in animal-based research. Although the error metrics highlighted the encouraging predictive performance of the foRcast model’s performance, its limitations were also evident. Abrupt changes during the animals’ observations violated the assumptions of stationarity and linearity, leading to greater deviations from the actual RELSA scores. To counteract these violations, we assumed high-resolution measurements to facilitate predictions via incremental changes rather than large deviations. Further, parameter volatility (e.g., in the activity) and increased data noise impede accurate predictions. Nevertheless, incorporating such an unsteady variable into the multivariate RELSA procedure could mitigate its impact, enabling better predictions. This study focused on humane endpoint predictions. However, scenarios with a peak in endured burden and subsequent improvement of the animals’ well-being will be predicted just as precisely if the improvement is seen in the outcome measures.

As the first work in this direction, we defined preliminary thresholds on the RELSA scale in a sepsis model to gain directly interpretable severity gradings. Because the RELSA procedure depends on the quality of its input variables, a lack of harmonized measurements across animal models impedes the development of a generalized RELSA scale. Assessing the same parameters using harmonized technical and methodological approaches across different experimental procedures, e.g., in an automated home cage, could pave the way for comparable datasets across different animal models and interventions and, subsequently, for a unified RELSA scale. Finally, this will allow more precise comparisons of burden and suffering across animal-based research in a severity map.

## Conclusion and outlook

The foRcast tool was designed to identify individual animals reaching humane endpoint criteria in time-series data. This study demonstrated, for the first time, the foRcast model’s potential for reliable, precise, and robust performance, thereby proving the concept for further analyses. Identifying humane endpoints is crucial in ethical animal-based research to prevent prolonged suffering, but it requires precise severity assessments. The RELSA procedure allows a multivariate, and therefore differentiated, severity assessment while paving the way for a general severity map. While demonstrating promising results, this work also highlighted the challenges in forecasting abrupt changes in animals’ well-being. With the further development of home cage monitoring systems, a computerized workflow for measuring, pre-processing, analyzing, and interpreting harmonized measurements will enable comparisons across many different animal models, prompting the definition of a generalized RELSA scale with defined thresholds for severity classes. In combination with the foRcast tool, experiments will be conducted and assessed simultaneously, allowing the handling personnel to give special attention to animals in need.

Overall, the foRcast model presents a valuable addition to the range of severity tools, helping refine and advance severity assessment in animal-based research.

## Data Availability

Publicly available datasets were analyzed in this study. This data can be found here: Sepsis and 1.5% DSS with restraint stress data: https://github.com/mytalbot/RELSA/tree/master/raw_data; DSS with repeated facial vein blood sampling: https://doi.org/10.1371/journal.pbio.2006159.s002; Pancreatic cancer: https://doi.org/10.1371/journal.pone.0261662; Neurosurgery: https://doi.org/10.6084/m9.figshare.26030569.
