# Supplementary material for: Refining humane endpoint detection by time-series forecasting and threshold definition using a multivariate severity score
Source: Front Physiol. 2026 Jul 8;17:1869563. doi: 10.3389/fphys.2026.1869563 (PMC13388068; doi:10.3389/fphys.2026.1869563)
Supplement: Supplementary file 1 [file DataSheet1.pdf]

Supplementary Material

S1 Comprehensive table of considered animal models.

Table S1: Comprehensive table of considered animal models and interventions, including treatment subgroups, strain, sex, number of animals used for the analyses, number of animals that have reached the humane endpoint, parameters used in the RELSA calculation, time point of reaching the humane endpoint, and criteria met for humane endpoint identification.

Animal model/ intervention	Treatment subgroups	Strain	Sex	Total No. of animals used for this analysis	No. of animals reaching humane endpoint	Outcome measures used for RELSA score calculation	Time point of reaching humane endpoint	Humane endpoint criteria met
Sepsis	CLP surgery	C57BL/6JN	♂	4	2	hr, hrv, temp, act	16 hours post surgery	>25% loss in temperature in more than two consecutive monitoring time points
	Sham surgery			3	-		-	-
DSS + Restraint Stress	1.5% DSS + Restraint Stress	C57BL/6J	♀	7	3	bwc, hr, hrv, temp, act	7 days after DSS administration	≥20% body weight loss
DSS + Blood Sampling	0% DSS	C57BL/6	♀	7	-	bwc, vwr	-	-
	1% DSS			8	-		-	-
	1.5% DSS			8	1		8 days after DSS administration	≥20% body weight loss

Animal model/ intervention	Treatment subgroups	Strain	Sex	Total No. of animals used for this analysis	No. of animals reaching humane endpoint	Outcome measures used for RELSA score calculation	Time point of reaching humane endpoint	Humane endpoint criteria met
	0% DSS + Phlebotomy			8	-		-	-
	1% DSS + Phlebotomy			14	4		6-9 days after DSS administration	Total clinical score of 5; Body weight loss of ≥20%
	1.5% DSS + Phlebotomy			9	2		9 days after DSS administration	≥20% body weight loss
	0% DSS + Restraint Stress			8	-		-	-
Pancreatic Cancer	Vehicle	C57BL/6J	♂	1	1	bwc, vwr	8 days post cell injection	≥20% body weight loss
Neurosurgery	Surgery + Carprofen treatment	C57BL6/J	♂	1	1	bwc, nest, neuro	Day 3 after surgery	Total clinical score of 7; Body weight loss of 11%; Hunched back and reduced spontaneous activity

Further details are reported in the original publications.

Table S2: Comprehensive table of variables used in the RELSA calculation, "turned" variables, baseline window, and reference group for all models.

Animal Model/Intervention	Input Variables	"Turned" variables	Baseline window	Reference group
Sepsis	hr, hrv, temp, act	-	All parameters: hour -1 before CLP surgery	Humane endpoint
1.5% DSS + Restraint Stress	bwc, hr, hrv, temp, act	hr, temp	bwc: day 0;<br>hr, hrv, temp, act: day 28 after transmitter implantation	1% DSS + Restraint Stress
DSS + Blood Sampling	bwc, vwr	-	bwc: day 0;<br>vwr: mean (day -3 – day -1)	1.5% DSS + Blood Sampling
Pancreatic Cancer	bwc, vwr	-	bwc: mean (day -3 – day -1);<br>vwr: mean (day -6 – day -1)	Vehicle
Neurosurgery	bwc, nest, neuro	nest, neuro	bwc: last measurement, day -3;<br>nest: day -3;<br>neuro: day -4	Carprofen monotreatment

S2 Evaluation and validation of the foRcast function.

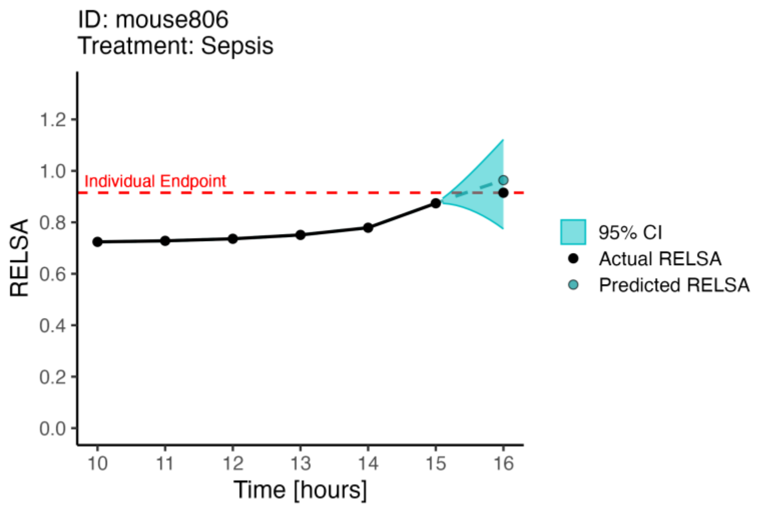

Figure S2: Individual RELSA score prediction of the humane endpoint of the mouse, which reached the humane endpoint in the sepsis model. The red dashed line depicts the RELSA scores at which the humane endpoint was reached. The black data points show the actual RELSA scores, while the blue data point represents the humane endpoint prediction. The blue ribbon outlines the 95% confidence intervals of the prediction. The foRcast prediction in this model achieved an RMSE of 0.009 and PICP of 100%.

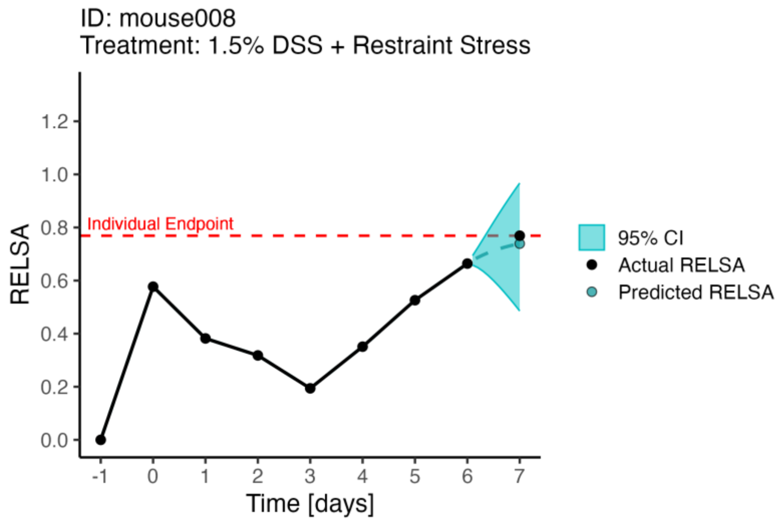

Figure S3: Individual RELSA score prediction of the mouse that reached the humane endpoint in the DSS model with restraint stress. The red dashed line depicts the RELSA scores at which the humane endpoint was reached. The black data points show the actual RELSA scores, while the blue data point represents the humane endpoint prediction. The blue ribbon outlines the 95% confidence intervals of the predictions. The foRcast predictions in this model achieved an RMSE of 0.007 and PICP of 100%.

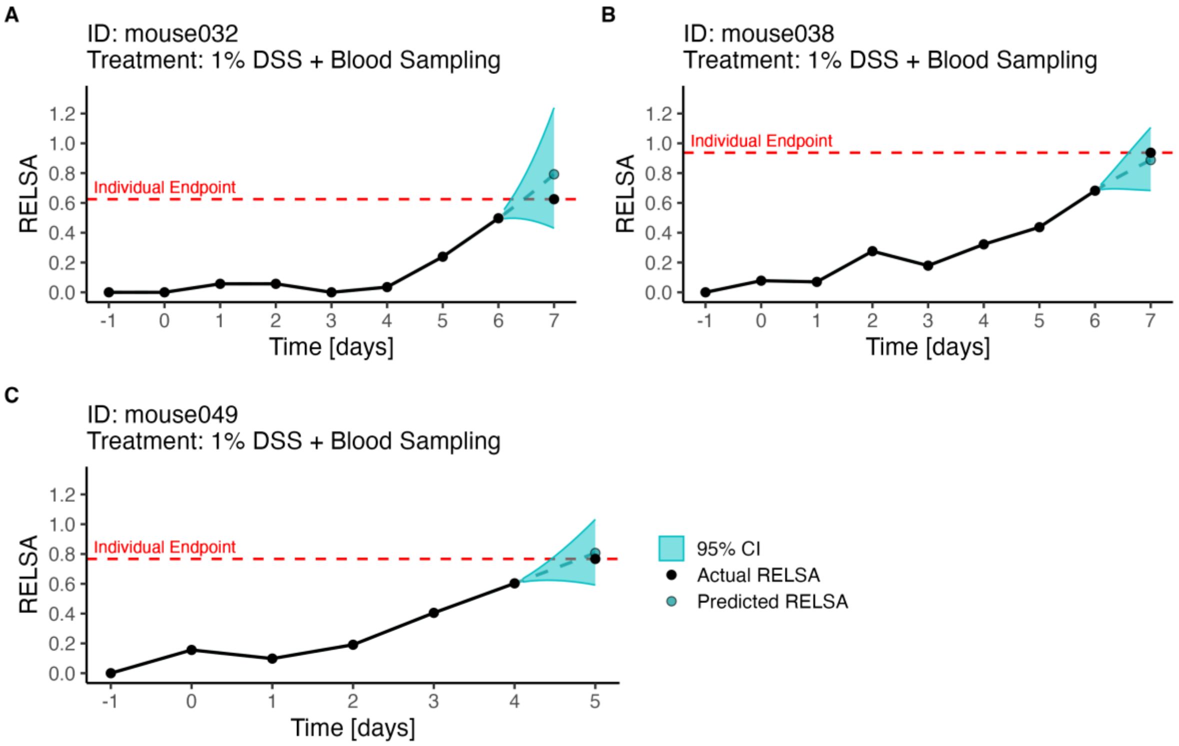

Figure S4: Individual RELSA score prediction of the pre-humane endpoint of the mice that reached the humane endpoint in the 1% DSS model and blood sampling condition. The red dashed lines depict the RELSA scores at which the humane endpoint was reached. The black data points show the actual RELSA scores, while the blue data points represent the humane endpoint prediction. The blue ribbons outline the 95% confidence intervals of the predictions. The foRcast predictions of all mice in this model achieved an RMSE of 0.046 and PICP of 75%.

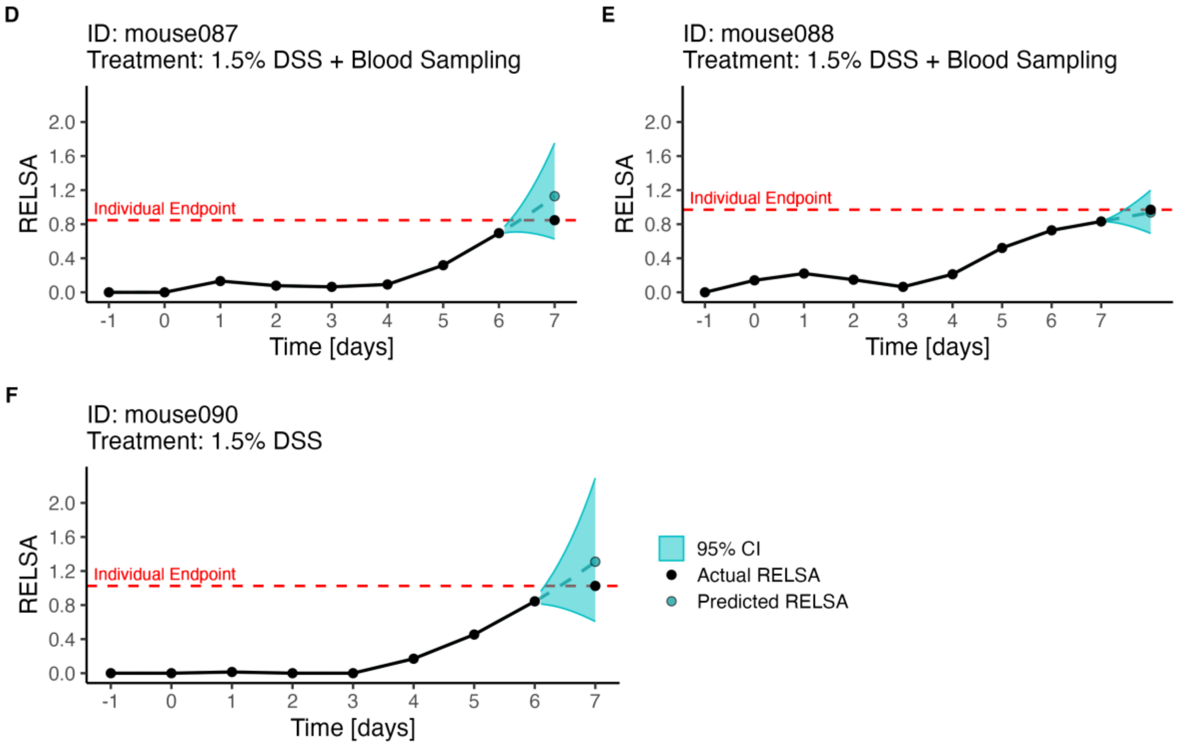

Figure S5: Individual RELSA score prediction of the pre-humane endpoint of the mice, which reached the humane endpoint in the 1.5% DSS model with blood sampling, and 1.5% DSS model without blood sampling. The red dashed lines depict the RELSA scores at which the humane endpoint was reached. The black data points show the actual RELSA scores, while the blue data points represent the humane endpoint prediction. The blue ribbons outline the 95% confidence intervals of the predictions. (E, F) The foRcast predictions for the mice in the 1.5% DSS model with blood sampling achieved an RMSE of 0.065 and a PICP of 100%. (G) The foRcast prediction of the single mouse that reached the humane endpoint in the 1.5% DSS model achieved an RMSE of 0.095 and PICP of 100%.

Table S3: Performance evaluation of the foRcast function with and without interpolation steps using RMSE, PICP, and MPIW per animal model and overall for mice that reached the humane endpoint.

Animal Model/Intervention	RMSE		PICP [%]		MPIW	
	With Interpolation	Without Interpolation	With Interpolation	Without Interpolation	With Interpolation	Without Interpolation
Sepsis	0.009	0.019	100	100	0.30	Infinite
1.5% DSS + Restraint Stress	0.007	0.081	100	50	0.66	0.98
1% DSS + Blood Sampling	0.046	0.052	75	75	0.53	1.08
1.5% DSS + Blood Sampling	0.065	0.047	100	100	0.84	Infinite
1.5% DSS	0.095	0.060	100	100	1.64	7.90
Pancreatic Cancer	0.177	0.323	100	100	7.35	30.59
Neurosurgery	0.082	0.043	100	100	0.54	2.41
Overall	0.069	0.089	96	89	1.69	-

S3 Definition of severity thresholds using kernel density estimation.

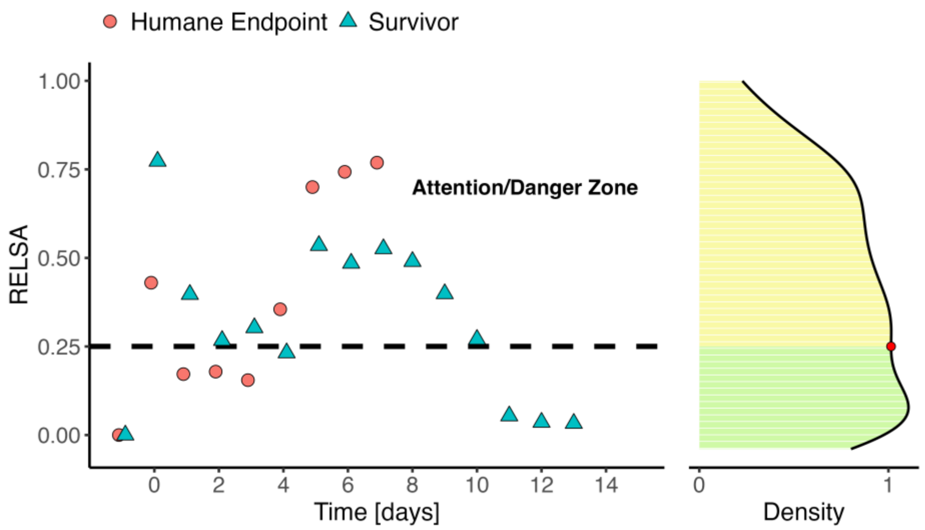

Figure S6: Definition of thresholds on the RELSA scale through kernel density estimation in the DSS model with restraint stress. Each data point shows the calculated RELSA course for an individual, exemplary animal at each time point, with red points indicating a mouse that reached the humane endpoint and blue triangles indicating a surviving mouse. The plot on the right shows the density curve with one minimum at RELSA = 0.250. Data from all animals in the DSS and restraint stress study were used in this analysis (n = 6 mice, 75 data points).

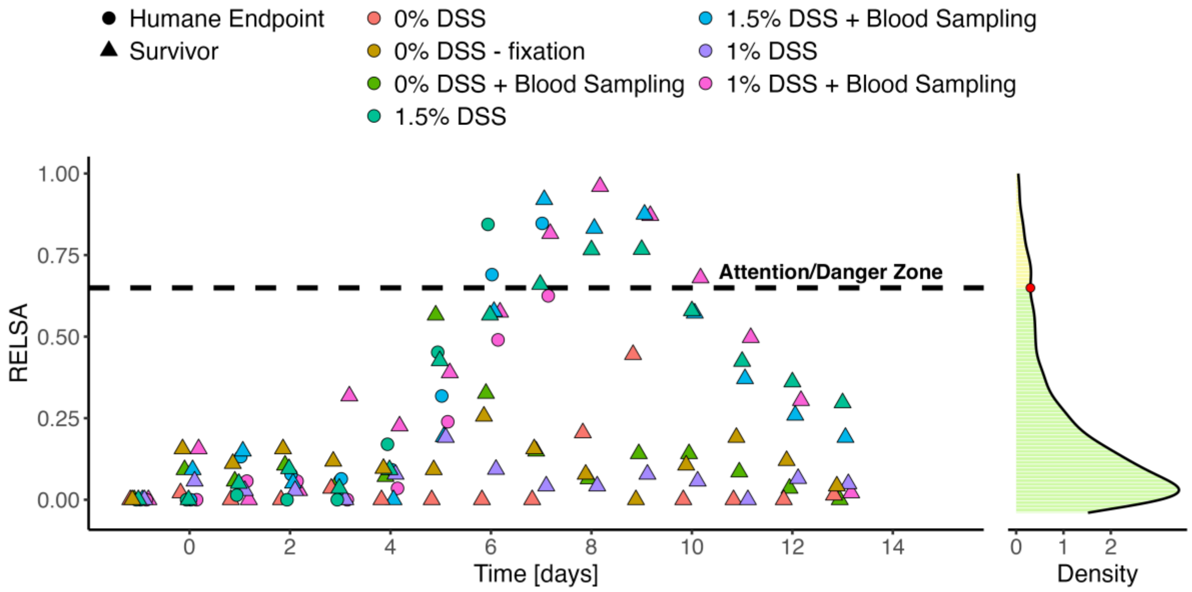

Figure S7: Definition of thresholds on the RELSA scale through kernel density estimation in the DSS model with blood sampling. Each data point shows the calculated RELSA course of an individual, exemplary animal at each time point, with points of different colors representing the treatment groups. Circles represent animal that reached the humane endpoint, while triangular data points represent surviving mice. The plot on the right shows the density curve with one minimum at RELSA = 0.649. Data from all animals in the DSS study with blood sampling were used in this analysis (n = 62 mice, 888 data points).

S4 Course of raw outcome measures of mice reaching humane endpoint criteria

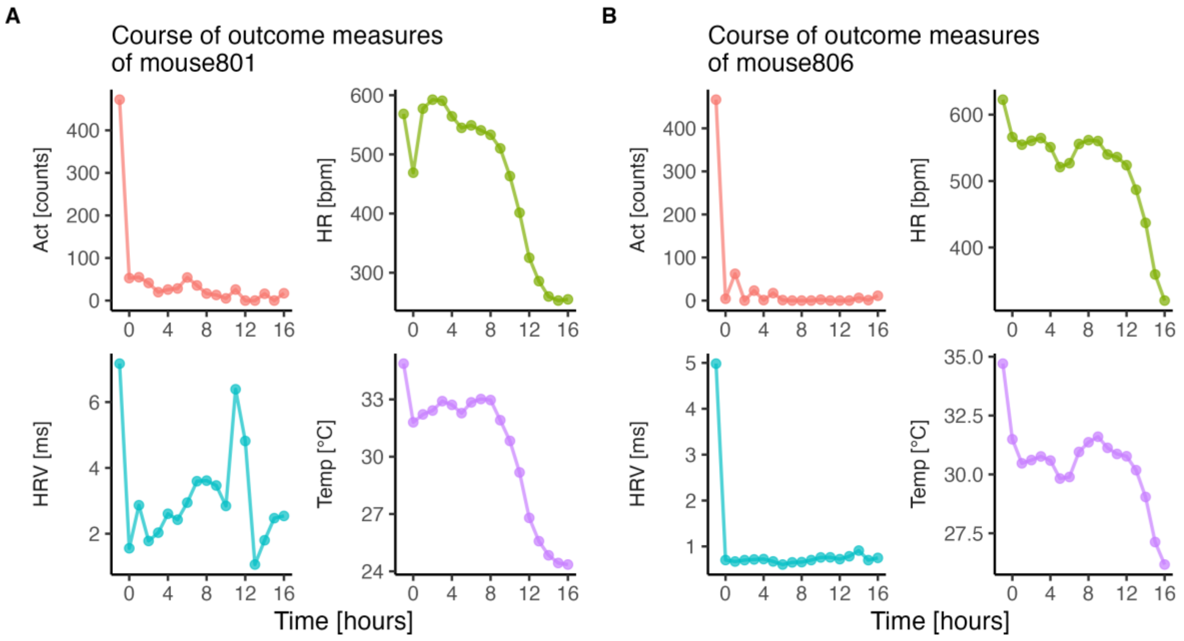

Figure S8: Course of raw outcome measures in the sepsis model of both mice that reached the humane endpoint. In the sepsis study, the telemetric parameters activity, heart rate, heart rate variability, and temperature were measured. (A) Course of the parameters in mouse 801, which reached the humane endpoint. (B) Course of the parameters in mouse 806, which reached the humane endpoint.

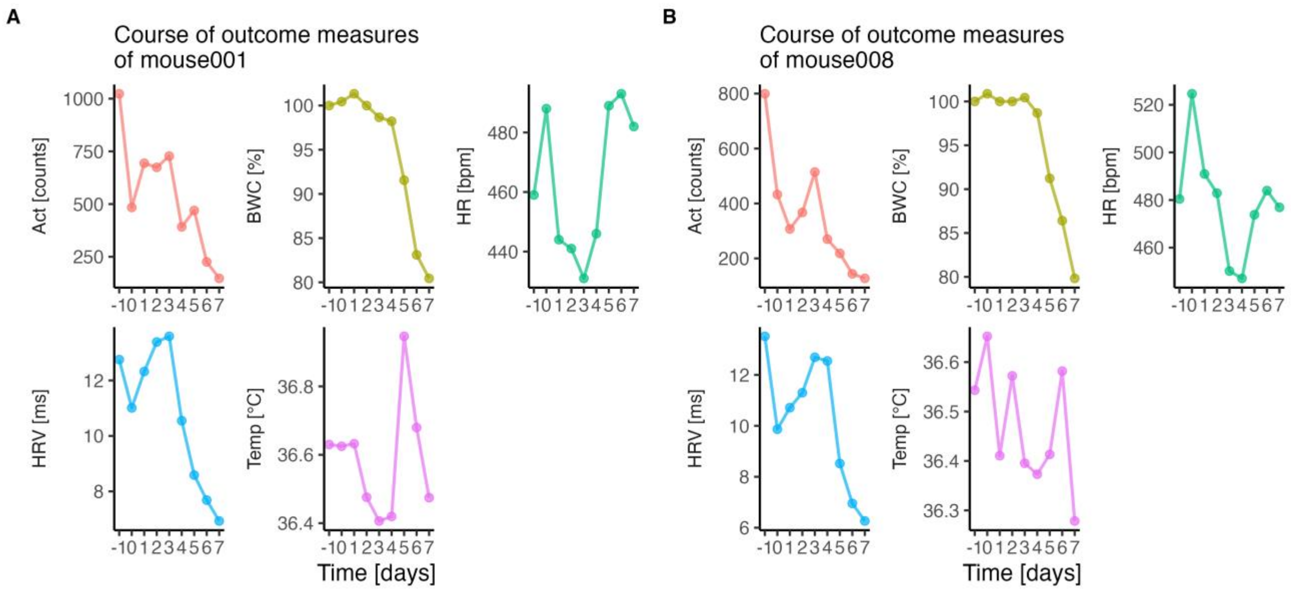

Figure S9: Course of raw outcome measures in the DSS model with restraint stress of both mice that reached the humane endpoint. In the DSS model with additional restraint stress, the telemetric parameters activity, heart rate, heart rate variability, and temperature were measured, as well as the body weight change. (A) Course of the parameters in mouse 001, which reached the humane endpoint. (B) Course of the parameters in mouse 008, which reached the humane endpoint.

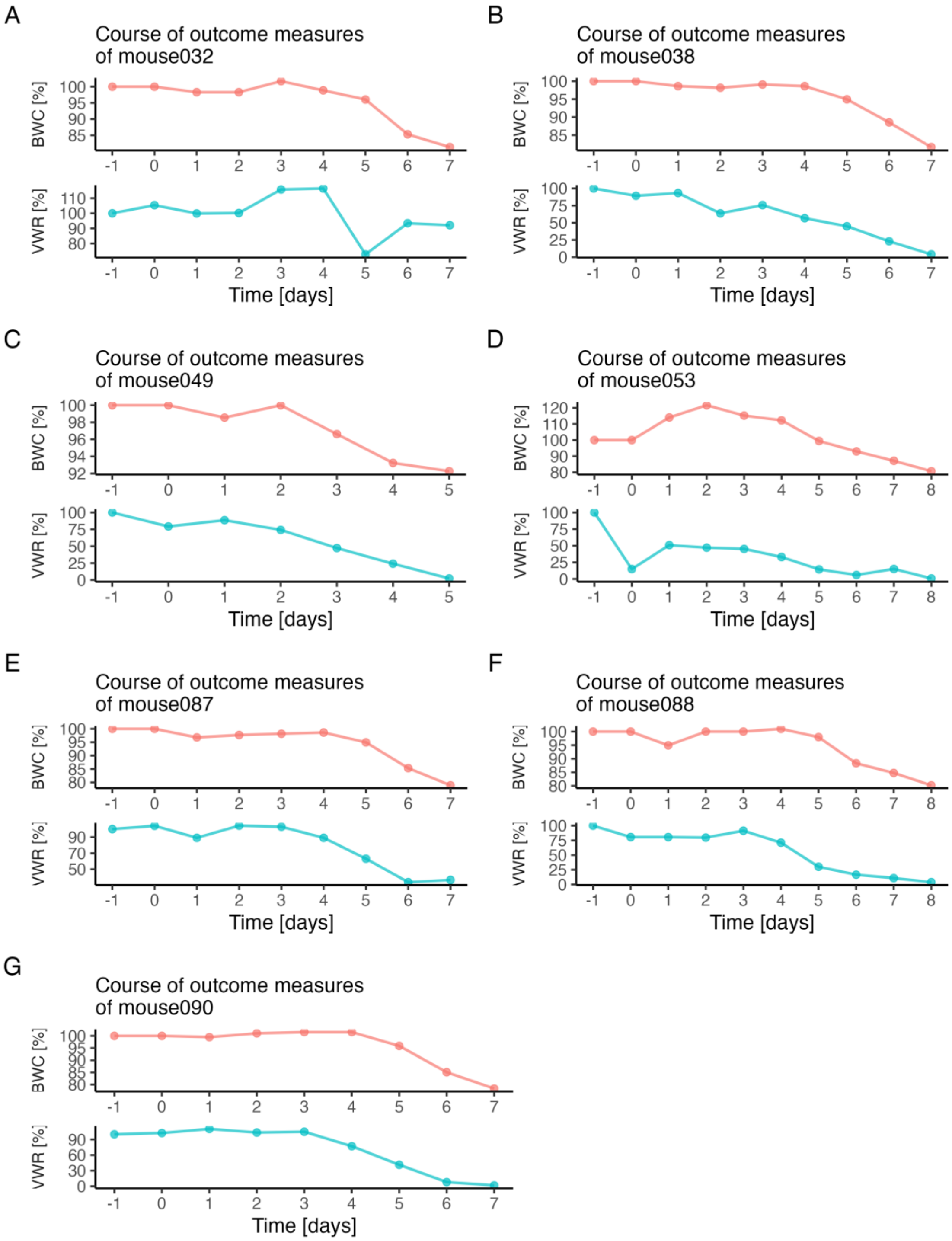

Figure S10: Course of raw outcome measures in the DSS model with blood sampling of the mice that reached the humane endpoint. In the DSS model with additional blood sampling, the parameters voluntary wheel running and body weight change were measured. (A) Course of the parameters in mouse 032, which reached the humane endpoint. (B) Course of the parameters in mouse 038, which reached the humane endpoint. (C) Course of the parameters in mouse 049, which reached the humane endpoint. (D) Course of the parameters in mouse 053, which reached the humane endpoint. (E) Course of the parameters in mouse 087, which reached the humane endpoint. (F) Course of the parameters in mouse 088, which reached the humane endpoint. (G) Course of the parameters in mouse 090, which reached the humane endpoint.

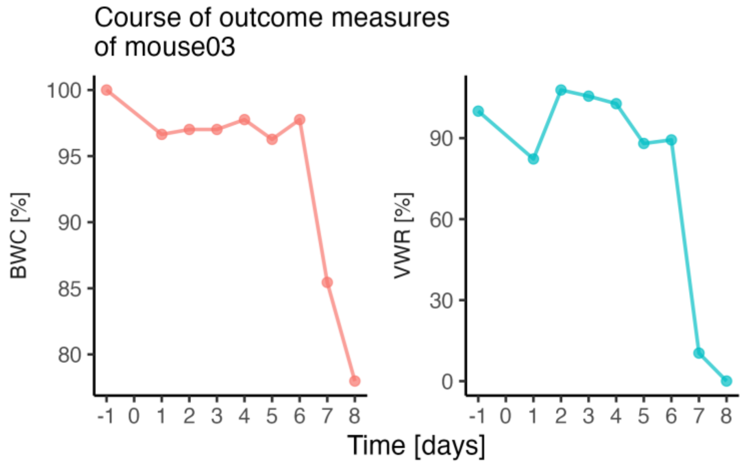

Figure S11: Course of raw outcome measures in the pancreatic cancer model of the single mouse that reached the humane endpoint. In the pancreatic cancer model, the parameters body weight change and voluntary wheel running were measured. Course of the parameters in mouse 03, which reached the humane endpoint.

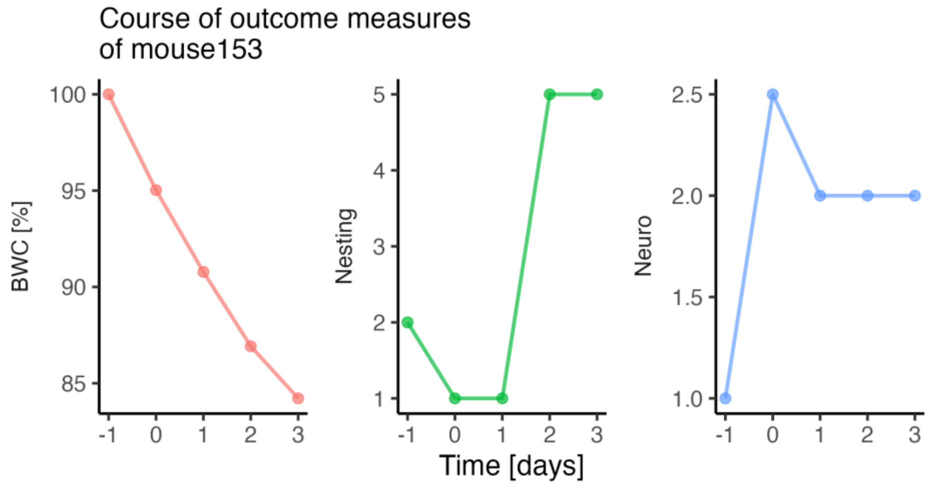

Figure S12: Course of raw outcome measures in the neurosurgical model of the single mouse that reached the humane endpoint. In the neurosurgical intervention, the parameters body weight change, nesting score, and Neuro score were measured. Course of the parameters in mouse 153, which reached the humane endpoint.
